# Identifying the Relative Importance of Factors Influencing Medication Compliance in General Patients Using Regularized Logistic Regression and LightGBM: Web-Based Survey Analysis

**DOI:** 10.2196/65882

**Published:** 2024-12-23

**Authors:** Haru Iino, Hayato Kizaki, Shungo Imai, Satoko Hori

**Affiliations:** 1 Division of Drug Informatics Faculty of Pharmacy and Graduate School of Pharmaceutical Sciences Keio University Tokyo Japan

**Keywords:** medication adherence, pharmacological management, medication compliance, Japan, drugs, dose, psychological, questionnaire survey, LightGBM, logistic regression model, regularization, machine learning, AI, artificial intelligence

## Abstract

**Background:**

Medication compliance, which refers to the extent to which patients correctly adhere to prescribed regimens, is influenced by various psychological, behavioral, and demographic factors. When analyzing these factors, challenges such as multicollinearity and variable selection often arise, complicating the interpretation of results. To address the issue of multicollinearity and better analyze the importance of each factor, machine learning methods are considered to be useful.

**Objective:**

This study aimed to identify key factors influencing medication compliance by applying regularized logistic regression and LightGBM.

**Methods:**

A questionnaire survey was conducted among 638 adult patients in Japan who had been continuously taking medications for at least 3 months. The survey collected data on demographics, medication habits, psychological adherence factors, and compliance. Logistic regression with regularization was used to handle multicollinearity, while LightGBM was used to calculate feature importance.

**Results:**

The regularized logistic regression model identified significant predictors, including “using the drug at approximately the same time each day” (coefficient 0.479; *P*=.02), “taking meals at approximately the same time each day” (coefficient 0.407; *P*=.02), and “I would like to have my medication reduced” (coefficient –0.410; *P*=.01). The top 5 variables with the highest feature importance scores in the LightGBM results were “Age” (feature importance 179.1), “Using the drug at approximately the same time each day” (feature importance 148.4), “Taking meals at approximately the same time each day” (feature importance 109.0), “I would like to have my medication reduced” (feature importance 77.48), and “I think I want to take my medicine” (feature importance 70.85). Additionally, the feature importance scores for the groups of medication adherence–related factors were 77.92 for lifestyle-related items, 52.04 for awareness of medication, 20.30 for relationships with health care professionals, and 5.05 for others.

**Conclusions:**

The most significant factors for medication compliance were the consistency of medication and meal timing (mean of feature importance), followed by the number of medications and patient attitudes toward their treatment. This study is the first to use a machine learning model to calculate and compare the relative importance of factors affecting medication adherence. Our findings demonstrate that, in terms of relative importance, lifestyle habits are the most significant contributors to medication compliance among the general patient population. The findings suggest that regularization and machine learning methods, such as LightGBM, are useful for better understanding the numerous adherence factors affected by multicollinearity.

## Introduction

Adherence to medication is an important component of pharmacological management, encompassing various factors, such as the relationship of the patient with the health care provider, individual behavior, and personal qualities [1-4]. Medication adherence is measured using a psychological factor scale that assesses the positive attitude of the patient toward treatment, as well as medication, and a scale that calculates the amount of medication taken [5]. On the other hand, since medication adherence assumes that the patient is in agreement, medication compliance is simply a more appropriate indicator of the extent to which the patient is taking the medication correctly [6,7]. Several quantitative measures of medication adherence exist, such as the medication possession rate, medication event monitoring system, and semiquantitative measures, which rely on self-reports [8-12]. Previous studies have documented numerous psychological adherence and risk factors associated with medication compliance [13-17]. However, these studies have not objectively assessed the significance of multiple factors related to medication adherence. Moreover, several analytical methods have encountered challenges.

The analysis of the association between the psychological factors of medication adherence and medication compliance commonly involved a regression analysis using a generalized linear model [18-21]. The response variable in this analysis was medication compliance. When dealing with multiple factors related to adherence, addressing variable selection and multicollinearity becomes necessary [22-25]. In clinical research, variable selection methods such as the filter method (using univariate analysis) and the stepwise method (using goodness-of-fit) are commonly used [26-29]. However, these methods do not consider the impact of variables as a group, and the selection of variables may vary depending on the starting time and the order of addition or removal [30,31]. In addition, because medication adherence is closely related to a patient’s treatment, multicollinearity may occur because of its inherent proximity [32]. Considering that multicollinearity can affect variable selection and increase covariates, this study uses two regularization terms (L1 and L2 norms), which have been used in genetic analyses when there are numerous dependent variables compared to the response variable [33-37]. This can automatically perform variable selection during training to handle challenges caused by multicollinearity [38-40].

The incorporation of explanatory variables into a first-order equation in generalized linear models presents limitations in expressing the relationship with the response variable [41]. To address this issue, we use a recently developed model, LightGBM, which combines multiple decision trees and offers the advantages of high accuracy and low computational cost [42,43]. Using this model, the contribution of each variable to the response variable can be quantified as feature importance during model construction, facilitating an objective understanding of the importance of factors. This study applies 2 machine learning approaches, logistic regression with regularization and LightGBM, to investigate the factors associated with medication compliance. These analyses overcome traditional challenges in exploring factors of medication adherence.

## Methods

### Questionnaire Survey

#### Survey Item Development

The questionnaire consisted of 4 main sections: patient background, medication-related items, psychological factors related to medication adherence, and medication compliance status. The patient characteristics included age, gender, medical conditions, and location. For medications used, patients were asked about the duration of medication use; the formulation and type of medication; and the type, dosage, and timing of medication intake throughout the day. Psychological factors for adherence were selected from those identified by Hiratsuka et al [44] and Ueno et al [45], and similar questions were asked to avoid duplication. HI and HK drafted the questions, and HI, HK, and SH conducted the final review. A total of 16 questions were asked regarding the psychological factors for adherence (Multimedia Appendix 1). Finally, four options were provided to ask about the details of noncompliance: (1) Never forget or skip to take medications, (2) Unintentionally forget to take medication (any frequency), (3) Intentionally skip to take medication (any frequency), and (4) Skip to take medication because I did not have medication when I intended to take it. These options were taken from a previous study by Hiratsuka et al [44] and were not found to correlate with the independent factors. In this study, option 1 was considered an exclusion, whereas the others were considered multiple-choice options. Important items, such as the distribution of participants in the questionnaire survey, are presented in the *Results* section of this paper, and other tabulated results are presented in the Multimedia Appendix 2.

#### Conducting a Survey

The survey was commissioned to INTAGE Inc, a Japanese market research company, which conducted it as an anonymous web-based questionnaire between November and December 2021. The questionnaire underwent a completeness check by the authors and INTAGE Inc, and the actual web interface was created. The questionnaire items were not randomized. The target population consisted of adults aged 20 years or older who had been taking their medication continuously for at least approximately 3 months. Only those who indicated in the screening survey that they had been taking their medication for at least three months were invited to participate. Respondents received redeemable points from the survey providers as compensation. INTAGE Inc has obtained JIS Y 20252 (ISO 20252), the international quality standard for market research, and appropriately excludes fraudulent responses.

### Constructing Machine Learning Models

#### Creating the Response Variable or Selecting the Model

For the noncompliance quality category, this study used a binary classification approach. Participants who chose “(1) Never forget or skip to take medications” were classified as the group adhering to medication correctly, while those who selected other options (2, 3, or 4) were considered as the group not adhering to medication correctly. Logistic regression and LightGBM were constructed with this as the response variable and other questionnaire items as explanatory variables. Based on the questionnaire, the characteristics of the participants’ backgrounds, medications, and lifestyles were constructed. Although the questionnaire contained 36 questions, some questions had one-hot expressions corresponding to the options, and finally, 64 variables were created (Multimedia Appendix 3).

#### Logistic Regression

The number of variables used in this study was 64, which surpasses the number of events in the response variable when performing logistic regression [46]. Hence, variable selection is necessary. In this study, variable selection was first performed based on univariate analysis (the filter method), and a logistic regression model was constructed as a filtered model. For univariate analysis, binary variables were subjected to the chi-square test or Fisher exact test (when the number of events was 10 or fewer cases per group). Likert scale responses were considered as continuous variables, and the Mann-Whitney *U* test was performed with features that were significantly different at the 5% confidence level (Multimedia Appendix 3).

As previously mentioned, the filter method has several problems. Therefore, as the second model in this study, we introduce an elastic-net-type model with regularization terms, which solves the drawbacks of the filter method [39,47]. This model uses two regularizations, the L1 and L2 norms, to perform variable selection during training but does not cut off variables excessively [47]. However, because the standard errors could not be calculated analytically using this regularization model, the bootstrap method was used to estimate the standard errors, and statistical tests were conducted [48-50].

However, covariates are possibly adjusted within groups of variables, and the explanatory power of individual variables in the model is distributed [51]. Therefore, to discuss multicollinearity and the importance of variables in the model, we calculate the variance inflation factor (VIF), a measure of multicollinearity, for both regularization and filter method models as a subanalysis and show the process of the cut-off of variables, a method to eliminate multicollinearity [52] (Multimedia Appendix 4). The variable with the highest VIF among the input variables was cut off, and the VIF was calculated again; this operation was repeated until the VIF of all variables was less than 10.

#### LightGBM

LightGBM can detect nonlinear relationships that cannot be identified by logistic regression through ensemble learning of decision trees. Additionally, LightGBM calculates feature importance, which allows us to quantitatively evaluate the relative impact of each variable on the model’s predictions. Furthermore, LightGBM includes a regularization function, enabling the analysis of data that contains variables with multicollinearity. For these reasons, we implemented LightGBM alongside logistic regression, as we believe it contributes to the robustness of this study’s results and enhances the interpretability of the importance of each factor.

LightGBM has many parameters that need to be tuned. In this study, Optuna, a package that uses the Tree-structured Parzen Estimator, was used as the tuning method [53,54]. A 5-fold cross-validation was performed for tuning. After determining the parameters, all data were fed into the final model, and feature importance was calculated. The gain, a type of feature importance that we used, is the sum of how much the accuracy of classification improves with the addition of branches in the decision tree for each feature. Feature importance has the same meaning as variable importance. To facilitate the interpretation of feature importance, the psychological factors affecting medication adherence were divided into four categories: (1) lifestyle-related items; (2) awareness of medication (acceptance, refusal, and expectations); (3) relationships with health care professionals; and (4) other items. The mean value of importance was calculated for each item.

### Ethical Considerations

This study complies with the Ethical Guidelines for Medical and Biological Research Involving Human Subjects published by the Ministry of Health, Labor, and Welfare of Japan, and all research plans were reviewed and approved by the Research Ethics Committee of the Keio University Faculty of Pharmacy (approval 211111-5). A web-based, unmarked questionnaire survey was used in this study. Informed consent was obtained from all participants by presenting them with an explanatory document and consent form prior to the survey administration. Only those who agreed to these documents were invited to participate in the survey. All procedures, including informed consent and the explanatory and consent documents presented to participants, were reviewed and approved by the Research Ethics Committee in compliance with ethical guidelines.

## Results

### Questionnaire Survey—Background of Participants

After the screening survey, 1000 individuals were invited to participate and 638 individuals completed the questionnaire. The demographic breakdown of the respondents was as follows: 68.8% (n=439) male and 31.2% (n=199) female. According to age group, 1.3% (n=8) were aged 20-29 years, 6.4% (n=41) aged 30-39 years, 13.2% (n=84) aged 40-49 years, 26.6% (n=170) aged 50-59 years, 27% (n=172) aged 60-69 years, 22.6% (n=144) aged 70-79 years, and 3% (n=19) aged 80-89 years or older (Table 1). The most prevalent diseases among respondents, accounting for more than 5% of the sample, were hypertension (n=169, 42.2%), hyperlipidemia (n=128, 20.1%), type 2 diabetes (n=84, 13.2%), constipation (n=55, 8.6%), psycho-nervous system disease (n=55, 8.6%), gastritis or gastroesophageal reflux disease (n=52, 8.2%), insomnia (n=38, 6%), and heart disease (n=32, 5%; Table 2). Regarding the duration of drug use, 2.7% (n=17) reported a period of 3 months to less than 6 months, 5% (n=32) for 6 months to less than 1 year, 16.3% (n=104) for 1 year to less than 3 years, and 76% (n=485) for 3 years or more (Table 3).

**Table 1:** Age distribution of participants (N=638).

Age group (years)	Female, n (%)	Male, n (%)	Total, n (%)
20s	8 (1.3)	0 (0)	8 (1.3)
30s	24 (3.8)	17 (2.7)	41 (6.4)
40s	38 (6)	46 (7.2)	84 (13.2)
50s	59 (9.2)	111 (17.4)	170 (26.6)
60s	43 (6.7)	129 (20.2)	172 (27)
70s	27 (4.2)	117 (18.3)	144 (22.6)
>80s	0 (0)	19 (3)	19 (3)
Total	199 (31.2)	439 (68.8)	638 (100)

**Table 2:** Disease distribution of respondents (N=638).

Disease	Value, n (%)
Type1 diabetes	9 (1.4)
Type 2 diabetes	84 (13.2)
Hypertension	269 (42.2)
Hyperlipidemia	128 (20.1)
Heart disease	32 (5)
Constipation	55 (8.6)
Gastritis or GERD^a^	52 (8.2)
IBD^b^	1 (0.2)
Rheumatoid arthritis	7 (1.1)
Asthma or COPD^c^	13 (2)
Allergic disease	27 (4.2)
Glaucoma	15 (2.4)
Insomnia	38 (6)
Psycho-nervous system disease	55 (8.6)
Kidney disease	3 (0.5)
Other disease	119 (18.7)

^a^GERD: gastroesophageal reflux disease.

^b^IBD: inflammatory bowel disease.

^c^COPD: chronic obstructive pulmonary disease.

**Table 3:** Duration of drug use (N=638).

Duration	Value, n (%)
≥3 months to <6 months	17 (2.7)
≥6 months to <1 year	32 (5)
≥1year to <3 years	104 (16.3)
≥3 years	485 (76)

### Logistic Regression

Results of the regularization model are presented in Table 4, whereas the results of the filter method model with feature selection using univariate analysis are shown in Table 5. Table S1 in Multimedia Appendix 4 presents the results of the univariate analysis. A total of 19 variables were selected in the regularized model, and 4 of them were found to be statistically significant: inflammatory bowel disease (IBD; *P*=.01), asthma or chronic obstructive pulmonary disease (COPD; *P*<.001), “Using the drug at approximately the same time each day” (*P*=.02), and “Taking meals at approximately the same time each day” (*P*=.02).

**Table 4:** Result of regularization model (logistic regression).

Features	Coefficient (95% CI)	*P* value
Type 1 diabetes	–1.97 (–4.65 to 0.845)	.10
Hyperlipidemia	–0.421 (–0.994 to 0.0532)	.09
IBD^a^	–12.0 (–26.5 to –1.16)	.01^b^
Asthma or COPD^c^	10.1 (8.15 to 12.3)	<.001^d^
I can share my thoughts and goals	0.410 (–0.110 to 0.853)	.15
Taking action to continue the medication	–0.390 (–0.959 to 0.232)	.23
Tablets or capsules (dosage forms used)	0.296 (–0.756 to 1.43)	.62
Eye drops (dosage forms used)	–0.621 (–1.31 to 0.0618)	.07
Others (dosage forms used)	–1.60 (–13.3 to 17.6)	.89
Not taking medication in the morning	0.206 (–0.581 to 1.09)	.59
Not using evening or nighttime medication	0.382 (–0.155 to 0.804)	.17
Anxious about taking medications	–0.113 (–0.398 to 0.217)	.44
I would like to have my medication reduced	–0.410 (–0.708 to –0.0746)	.01^b^
Taking medication is part of my lifestyle, like eating or brushing my teeth	0.0883 (–0.206 to 0.322)	.74
I take the same number and frequency of medicines every day	0.0937 (–0.131 to 0.419)	.38
Using the drug at approximately the same time each day	0.479 (0.0613 to 0.772)	.02^b^
Taking meals at approximately the same time each day	0.407 (0.09 to 0.765)	.02^b^
Number of drugs prescribed (morning)	0.261 (–0.0269 to 0.551)	.08
Number of drugs prescribed (before bedtime)	–0.106 (–0.332 to 0.115)	.34

^a^IBD: inflammatory bowel disease.

^b^*P*<.05.

^c^COPD: chronic obstructive pulmonary disease.

^d^*P*<.01.

**Table 5:** Filter method model (logistic regression).

Features	Coefficient (95% CI)	*P* value
Type 1 diabetes	–1.67 (–3.18 to –0.154)	.03^a^
Hypertension	0.0517 (–0.402 to 0.505)	.82
Asthma or COPD^b^	25.0 (–83700 to 83800)	≥.99
I can share my thoughts and goals	0.380 (–0.041 to 0.802)	.08
Eating three meals every day	–0.383 (–1.25 to 0.487)	.39
Sometimes don’t eat breakfast	–0.143 (–1.08 to 0.795)	.77
Tablets or capsules (dosage forms used)	0.489 (–0.446 to 1.42)	.31
Inhaler (dosage forms used)	–0.628 (–3.00 to 1.75)	.60
Not taking medication in the morning	–0.0376 (–0.855 to 0.780)	.93
Taking medicines after breakfast	–0.0698 (–0.637 to 0.497)	.81
Age	–0.0009 (–0.019 to 0.017)	.92
No evening or nighttime medication	0.405 (–0.06 to 0.87)	.09
Duration of using drug	–0.0486 (–0.341 to 0.244)	.74
I’m convinced of the necessity of medicine	–0.142 (–0.498 to 0.214)	.43
I think I can’t stay healthy without medication	0.014 (–0.238 to 0.266)	.91
I think I want to go off my medicine	0.002 (–0.236 to 0.240)	.99
Anxious about taking medication	–0.130 (–0.362 to 0.102)	.27
I would like to have my medication reduced	–0.355 (–0.608 to –0.101)	.006^c^
Taking medication is part of my lifestyle, like eating and brushing my teeth	0.144 (–0.144 to 0.432)	.33
Take the same number and frequency of medicines every day	0.134 (–0.154 to 0.422)	.36
Using the drug at approximately the same time each day	0.471 (0.113 to 0.828)	.01^a^
Taking meals at approximately the same time each day	0.514 (0.194 to 0.834)	.002^c^
Number of drugs prescribed (morning)	0.106 (–0.078 to 0.291)	.26
Number of drugs prescribed (before bedtime)	–0.156 (–0.402 to 0.090)	.21

^a^*P*<.05.

^b^COPD: chronic obstructive pulmonary disease.

^c^*P*<.01.

The process of constructing a logistic regression model with multicollinearity eliminated is detailed in Multimedia Appendix 4, where Table S1 presents the regularization model variable and Table S2 shows the process with the filter method variable. The VIF is available in Tables S3 and S4 in Multimedia Appendix 4. In the filter model, 24 variables were selected, and 4 of them were found to be statistically significant: type 1 diabetes (*P*=.03), “I would like to have my medication reduced” (*P*=.006), “Using the drug at approximately the same time each day” (*P*=.01), and “Taking meals at approximately the same time each day” (*P*=.002).

### LightGBM

**Figure 1:**
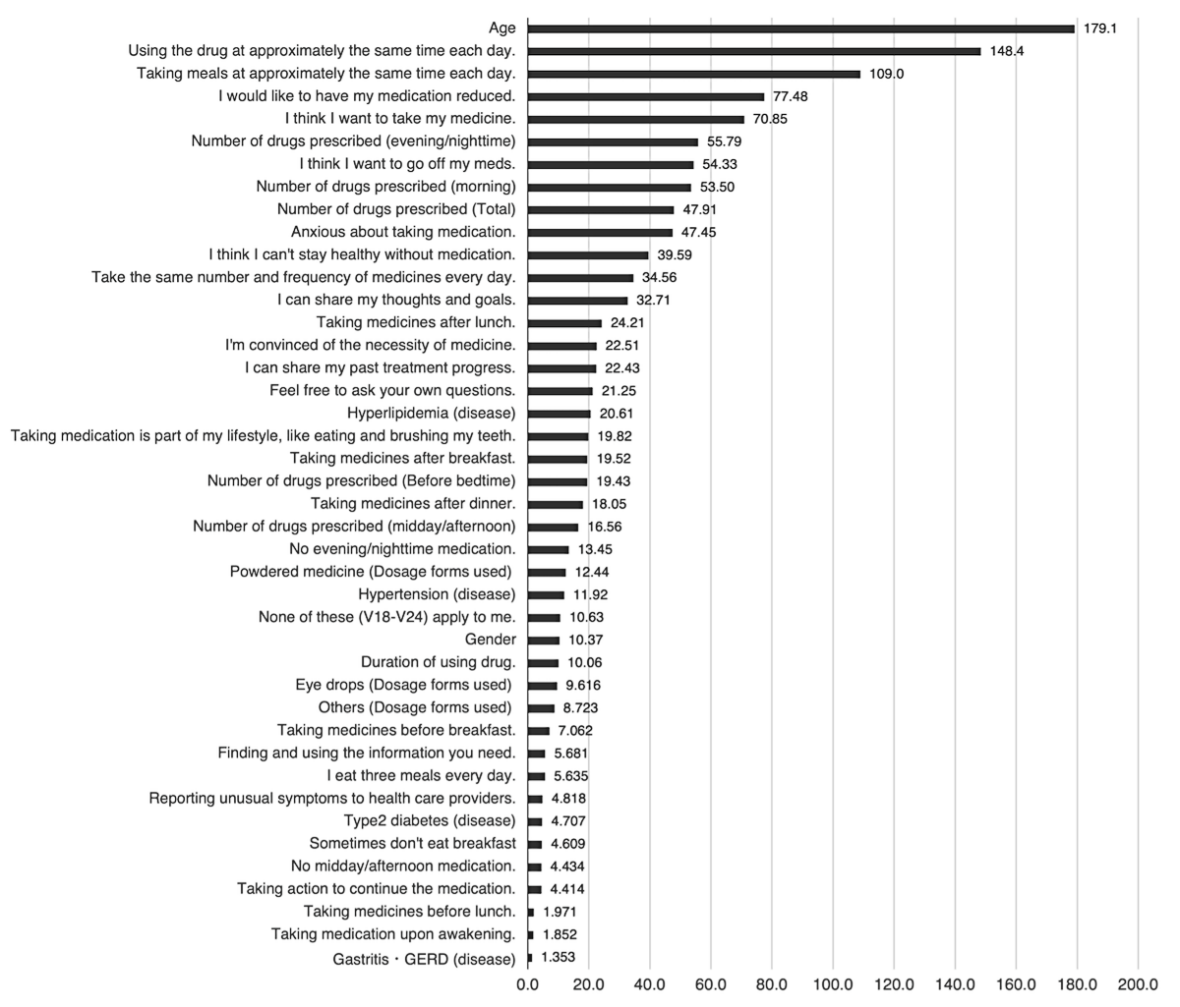
Feature Importance for each variable. A total of 42 variables were selected by the model, and the feature importance for each was calculated. Each bar represents the magnitude of the feature importance, and the numerical values indicate the actual calculated feature importance. GERD: gastroesophageal reflux disease.

The results of the feature importance calculation using LightGBM are displayed in Figure 1. The top 5 variables with the highest feature importance scores were “Age,” “Using the drug at approximately the same time each day,” “Taking meals at approximately the same time each day,” “I would like to reduce my medication,” and “I think I want to take my medicine.”

## Discussion

### Principal Findings

In this study, we conducted a questionnaire-based survey of medication adherence factors and compliance in the general patient population in Japan and used multiple models to determine their associations. While numerous factors related to medication adherence have been suggested previously, the relative importance of these items has not been demonstrated. In this study, we presented the relative importance of medication adherence factors through results such as feature importance and Table 6. The respondents were divided into two categories: one consisted of individuals who were taking their medication correctly, while the other included those who were not. Subsequently, two logistic regression analyses were conducted. Two characteristics showed significant differences were “Using the drug at approximately the same time each day” and “Taking meals at approximately the same time each day.” In the LightGBM, these two items were the second and third most common, similar to the results of the logistic regression analysis.

**Table 6:** The rank and mean of feature importance for medication adherence-related factors.

Groups of medication adherence–related factors and medication adherence–related psychological factors		Rank of feature importance	Rank of feature importance among psychological factors	Feature importance	Mean of feature importance within group	
**Lifestyle-related items**						77.95
	Using the drug at approximately the same time each day	2	1	148.4		
	Taking meals at approximately the same time each day	3	2	109.0		
	Take the same number and frequency of medicines every day	12	8	34.56		
	Taking medication is part of my lifestyle, like eating and brushing my teeth	19	13	19.82		
**Awareness of medication (acceptance, refusal, and expectations)**						52.04
	I would like to have my medication reduced	4	3	77.48		
	I think I want to take my medicine	5	4	70.85		
	I think I want to go off my medicine	7	5	54.33		
	Anxious about taking medication	10	6	47.45		
	I think I can’t stay healthy without medication	11	7	39.59		
	I’m convinced of the necessity of medicine	15	10	22.51		
**Relationship with health care professional**						20.30
	I can share my thoughts and goals	13	9	32.71		
	I can share my past treatment progress	16	11	22.43		
	Feel free to ask your own questions	17	12	21.25		
	Reporting unusual symptoms to health care providers.	35	15	4.818		
**Others**						5.05
	Finding and using the information you need	33	14	5.681		
	Taking action to continue the medication	39	16	4.414		

This is the first study to use a machine learning model to calculate the importance of factors related to medication compliance, which is important in determining intervention priorities. In this study, age, acceptance or refusal of medication, and number of medications taken were identified as important characteristics of feature importance. By calculating feature importance, we can quantitatively demonstrate the relative impact of each factor on the model’s predictions. This enhances the interpretability of the analysis and is useful for clinical applications such as prioritizing interventions.

In addition, some features, such as age, were not significantly different in the logistic regression; however, it ranked high in feature importance in LightGBM. The linear predictors in the generalized linear model are fixed at first-order expressions. This can be challenging in handling cases with explanatory variables, which are represented by polynomials of second- or higher-order, or special functions. Therefore, these features are likely to exhibit nonlinear relationships. Previous studies have shown that medication adherence improves with increasing age for many diseases; however, it declines after the age of 70 years due to the effects of cognitive decline [24,55]. Negative effects have also been observed in some diseases; however, these demonstrate a nonlinear age-related relationship. These results indicate that age is one of the most important factors in medication compliance and that age-related interventions in clinical practice can be effective [56,57].

The rank order and value of feature importance are presented for the psychological factors of medication adherence, and the mean of feature importance was calculated for each group (Table 6). The items related to the awareness of medication (acceptance, refusal, and expectation), lifestyle-related items, and other items were approximately in the same rank order, and the feature importance values deviated from each other by a factor of more than 2. Although whether the bottom two variables in the lifestyle-related items were far apart from the top two variables is unclear, the mean feature importance indicated that the psychological factors of medication compliance and related adherence were (1) lifestyle-related items, (2) awareness of medication (acceptance, refusal, and expectation), (3) relationships with health care professionals, and (4) others, in order of importance. To the best of our knowledge, this is the first study to calculate and discuss the relative significance of each medication adherence factor in terms of feature importance in the general patient population [20].

Comparing the prediction accuracy of the models created in this study, the area under the curve, an evaluation index of prediction accuracy, improved from 0.69 for normal models to 0.76 for regularization models. In addition, a comparison of the calculated coefficients and 95% CIs for asthma and COPD suggests that the regularization terms were effective in suppressing the overestimation of the coefficient. In addition, because regularization also has the effect of suppressing overfitting, the appropriate variable selection is presumed to lead to an improvement in accuracy [58].

In regularization models, the underestimation of partial correlation coefficients within multicollinearity groups can cause missing variables that are relevant when significance is the criterion [59,60]. Multimedia Appendix 4 shows the results of creating a normal logistic regression model from the variables selected in the two models and further reducing the variables until multicollinearity was solved based on the VIF. When significant variables were removed, other variables became significant. Although the mathematical basis is unclear, the order of the variables in the LightGBM results was almost identical to that in the logistic regression analysis, suggesting that the important variables in the model were shifting. For instance, for traits such as “Anxious about taking medication” where the tendency of the trait was similar to that of the other traits, and the explanatory power in the model was lost to the other variables. However, by removing the other variables, the explanatory power that should have been carried by that variable was demonstrated.

The variables selected by the filter method are presented as results after they were removed based on the VIF. Unlike the regularization model, variables that became significant sometimes ceased to be significant in the process of resolving multicollinearity, indicating that the model was not stable. This is because the filter method selects noisy variables, and variable selection using the regularization method may be useful for selecting variables with complex relationships, such as confounders.

### Limitations

First, regarding external validity, the population surveyed in this study was recruited from patients registered on an internet panel, and any deviation from the actual demographics may have affected the results [61]. The results of this study may be influenced by Japan’s cultural and social background and health care system. Additionally, because the survey was conducted using the internet, older people and those who do not use the internet may have been underrepresented, potentially leading to sampling bias. The differences between our sample and the actual demographics in Japan are discussed below. Therefore, we compared the present population with statistical information published by the Japanese government [62]. No major differences were found in terms of gender and disease rates. However, in terms of age groups, the number of patients in the age group above the late 60s was smaller than the actual demographics. This may be due to a decrease in the number of participants due to barriers to internet access in the case of those older than 60 years. In addition, the random recruitment in this study limits us to collect sufficient data for the analysis of diseases with low incidence rates. In particular, two variables entered into the two logistic regression models in the current analysis—IBD and asthma or COPD—had statistically unwieldy values, with a count of zero in one group when the response variable was classified into two groups (Multimedia Appendix 3). The coefficients and CIs diverged for these two variables, which may have affected the other variables [63]. Therefore, we recreated a model without these two variables (Multimedia Appendix 5). In the regularization model, 15 of the 17 variables were equal and the statistically significant variables remained the same, except for eye drops (dosage forms used), which was newly significant. Most of the variables that ranked high in LightGBM feature importance remained consistent despite these changes, suggesting that the impact of IBD and asthma or COPD on the overall model is likely limited. However, a new variable selection “I think I want to go off my medicine” emerged as one of the top variables in LightGBM feature importance after removing the variables. The elimination of the two anomalous variables might have enabled the correct variable selection.

### Conclusions

The most important factor influencing medication compliance was consistent with the timing of medication intake and meal consumption. The subsequent factors were the number of medications taken and feelings of acceptance or refusal of medication. Although these factors have been mentioned in previous studies, we were able to calculate their importance using a machine learning model. Few studies have mentioned adherence and lifestyles of patients, and further research could shed light on medication adherence in terms of daily behaviors of people.

In addition, when adherence factors are used as features, multicollinearity may be generated because of similarities in their respective characteristics. Therefore, caution should be exercised when discussing the relationship between response variables using generalized linear models. When multicollinearity is addressed, examining the relevance or considering alternative models, such as regularization or decision trees, that can effectively handle the issue of multicollinearity is important.

## Appendix

Multimedia Appendix 1 16 Items of adherence-related factors (psychological factors).

Multimedia Appendix 2 Detailed results of the patient questionnaire survey.

Multimedia Appendix 3 Results of the univariate analysis.

Multimedia Appendix 4 The process of constructing a logistic regression model.

Multimedia Appendix 5 Result of the logistic regression without complete separation.

## Abbreviations

COPD: chronic obstructive pulmonary disease
IBD: inflammatory bowel disease
VIF: variance inflation factor

## Data Availability

The datasets generated during or analyzed during this study are not publicly available due to ethical considerations but are available from the corresponding author upon reasonable request.
