# Supplementary material for: Identifying the Relative Importance of Factors Influencing Medication Compliance in General Patients Using Regularized Logistic Regression and LightGBM: Web-Based Survey Analysis
Source: JMIR Form Res. 2024 Dec 23;8:e65882. doi: 10.2196/65882 (PMC11704655; doi:10.2196/65882)
Supplement: Multimedia Appendix 1 [file formative_v8i1e65882_app1.docx]

|  | How to respond |
| --- | --- |
| I can share my thoughts and goals. | Yes/No |
| I can share my past treatment progress. | Yes/No |
| Feel free to ask your own questions. | Yes/No |
| Finding and using the information you need. | Yes/No |
| Taking action to continue the medication. | Yes/No |
| Reporting unusual symptoms to health care providers. | 5-Level |
| I'm convinced of the necessity of medicine. | 5-Level |
| I think I can't stay healthy without medication. | 5-Level |
| I think I want to take my medicine. | 5-Level |
| I think I want to go off my medicine. | 5-Level |
| Anxious about taking medication. | 5-Level |
| I would like to have my medication reduced. | 5-Level |
| Taking medication is part of my lifestyle, like eating and brushing my teeth. | 5-Level |
| Take the same number and frequency of medicines every day. | 5-Level |
| Using the drug at approximately the same time each day. | 5-Level |
| Taking meals at approximately the same time each day. | 5-Level |
