# Supplementary material for: Identifying the Relative Importance of Factors Influencing Medication Compliance in General Patients Using Regularized Logistic Regression and LightGBM: Web-Based Survey Analysis
Source: JMIR Form Res. 2024 Dec 23;8:e65882. doi: 10.2196/65882 (PMC11704655; doi:10.2196/65882)
Supplement: Multimedia Appendix 2 [file formative_v8i1e65882_app2.docx]

Table S1. About the psychological factors of medication adherence (yes/no questionnaires)

| Psychological factors of medication adherence（Y/N） | | |
| --- | --- | --- |
|  | Yes | No |
| I can share my thoughts and goals. | 296(46.4%) | 342(53.6%) |
| I can share my past treatment progress. | 331(51.9%) | 307(48.1%) |
| Feel free to ask your own questions. | 344(53.9%) | 294(46.1%) |
| Finding and using the information you need. | 167(26.2%) | 471(73.8%) |
| Taking action to continue the medication. | 136(21.3%) | 502(78.7%) |
| Reporting unusual symptoms to health care providers. | 123(19.3%) | 515(80.7%) |

Table S2. About the Psychological factors of medication adherence (Likert-scale questionnaires)

| Psychological factors（5-level） | | |  |  |  |
| --- | --- | --- | --- | --- | --- |
|  | 1(Strongly agree) | 2（Agree） | 3（Neither agree nor disagree） | 4（Disagree） | 5（Strongly disagree） |
| I'm convinced of the necessity of medicine. | 269(42.2%) | 304(47.6%) | 57(8.9%) | 6(0.9%) | 2(0.3%) |
| think I can't stay healthy without medication. | 125(19.6%) | 224(35.1%) | 196(30.7%) | 84(13.2%) | 9(1.4%) |
| I think I want to take my medicine. | 96(15.0%) | 195(30.6%) | 180(28.2%) | 126(19.7%) | 41(6.4%) |
| I think I want to go off my medicine. | 67(10.5%) | 186(29.2%) | 228(35.7%) | 100(15.7%) | 57(8.9%) |
| Anxious about taking medication. | 25(3.9%) | 130(20.4%) | 192(30.1%) | 214(33.5%) | 77(12.1%) |
| I would like to have my medication reduced. | 62(9.7%) | 189(29.6%) | 219(34.3%) | 129(20.2%) | 39(6.1%) |
| Taking medication is part of my lifestyle, like eating and brushing my teeth. | 183(28.7%) | 319(50.0%) | 98(15.4%) | 31(4.9%) | 7(1.1%) |
| Take the same number and frequency of medicines every day. | 315(49.4%) | 249(39.0%) | 43(6.7%) | 22(3.4%) | 9(1.4%) |
| Using the drug at approximately the same time each day. | 280(43.9%) | 268(42.0%) | 59(9.2%) | 23(3.6%) | 8(1.3%) |
| Taking meals at approximately the same time each day. | 252(39.5%) | 272(42.6%) | 70(11.0%) | 34(5.3%) | 10(1.6%) |

Table S3．Dietary intake

|  | n= |
| --- | --- |
| I eat three meals every day. | 488(76.5%) |
| Sometimes don't eat breakfast | 113(17.7%) |
| Sometimes don't eat lunch | 49(7.7%) |
| Sometimes don't eat dinner | 16(2.5%) |

Table S4. Number of kind of medications used (All day).

|  | n= |
| --- | --- |
| 1 | 152(23.8%) |
| 2 | 145(22.7%) |
| 3 | 114(17.9%) |
| 4-5 | 127(19.9%) |
| 6-8 | 75(11.8%) |
| More than 9 | 25(3.9%) |

Table S5. The dosage form of the drug you are using.

| Dosage form | n= |
| --- | --- |
| Tablets/Capsules | 609(95.5%) |
| Powdered medicine | 78(12.2%) |
| Tape, Poultice, Plaster | 33(5.2%) |
| Inhaler | 17(2.7%) |
| Ointment | 40(6.3%) |
| Injection | 14(2.2%) |
| Eye drops | 66(10.3%) |
| Nasal drops | 19(3.0%) |
| Others | 5(0.8%) |

Table S6 Timing of medication use

|  | n= |
| --- | --- |
| Morning |  |
| Upon awakening | 44(6.9%) |
| After breakfast | 464(72.7%) |
| Before breakfast | 100(15.7%) |
| Midday/Afternoon |  |
| After lunch | 160(25.1%) |
| Before lunch | 55(8.6%) |
| Evening/Nighttime |  |
| After dinner | 321(50.3%) |
| Before dinner. | 71(11.1%) |
| Before bedtime | 152(23.8%) |

Table S7. Number of kind of medications used (Each timing)

| Morning | n= |
| --- | --- |
| 1 | 169(26.5%) |
| 2 | 145(22.7%) |
| 3 | 97(15.2%) |
| 4-5 | 94(14.7%) |
| 6-8 | 44(6.9%) |
| More than 9 | 9(1.4%) |
| Midday/Afternoon |  |
| 1 | 118(18.5%) |
| 2 | 42(6.6%) |
| 3 | 23(3.6%) |
| 4-5 | 13(2.0%) |
| 6-8 | 4(0.6%) |
| More than 9 | 1(0.2%) |
| Evening/Nighttime |  |
| 1 | 157(24.6%) |
| 2 | 120(18.8%) |
| 3 | 70(11.0%) |
| 4-5 | 54(8.5%) |
| 6-8 | 22(3.4%) |
| More than 9 | 5(0.8%) |
| Before bedtime |  |
| 1 | 94(14.7%) |
| 2 | 34(5.3%) |
| 3 | 10(1.6%) |
| More than 4 | 14(2.2%) |

Table S8. Medication compliance status

|  | n= |
| --- | --- |
| Never forget or skip to take medications. | 464(72.7%) |
| Unintentionally forget to take medication (Any frequency). | 72(11.3%) |
| Intentionally skip to take medication (Any frequency). | 92(14.4%) |
| Skip to take medication because I did not have medication when intend to take it. | 22(3.4%) |
