# Supplementary material for: Identifying the Relative Importance of Factors Influencing Medication Compliance in General Patients Using Regularized Logistic Regression and LightGBM: Web-Based Survey Analysis
Source: JMIR Form Res. 2024 Dec 23;8:e65882. doi: 10.2196/65882 (PMC11704655; doi:10.2196/65882)
Supplement: Multimedia Appendix 3 [file formative_v8i1e65882_app3.docx]

S3 Response variables & Results of univariate analysis.

The 64 items shown in 3-1 and 3-2 were used as response variables in the machine learning model of this study.

- 1. Univariate analysis: binary variables (chi-square test or Fisher's exact test (when the number of events was 10 or fewer cases per group).

| Variables | Taking the medication correctly | Not taking the medication correctly | p-value |
| --- | --- | --- | --- |
| Type1 diabetes (disease) | 5(0.8%) | 4(0.6%) | 0.067 |
| Type2 diabetes (disease) | 24(3.8%) | 60(9.4%) | 0.774 |
| Hypertension (disease) | 64(10.0%) | 205(32.1%) | 0.092 |
| Hyperlipidemia (disease) | 39(6.1%) | 89(13.9%) | 0.364 |
| Heart disease (disease) | 6(0.9%) | 26(4.1%) | 0.267 |
| Constipation (disease) | 16(2.5%) | 39(6.1%) | 0.752 |
| Gastritis・GERD (disease) | 13(2.0%) | 39(6.1%) | 0.701 |
| IBD (disease) | 1(0.2%) | 0(0.0%) | 0.273 |
| RA (disease) | 1(0.2%) | 6(0.9%) | 0.680 |
| Asthma・COPD (disease) | 0(0.0%) | 13(2.0%) | 0.024 |
| Allergic disease (disease) | 8(1.3%) | 19(3.0%) | 0.779 |
| Glaucoma (disease) | 6(0.9%) | 9(1.4%) | 0.254 |
| Insomnia (disease) | 14(2.2%) | 24(3.8%) | 0.172 |
| Psycho-nervous system disease (disease) | 18(2.8%) | 37(5.8%) | 0.342 |
| Kidney disease (disease) | 1(0.2%) | 2(0.3%) | 1.000 |
| Other disease | 31(4.9%) | 88(13.8%) | 0.820 |
| [V18] I can share my thoughts and goals. | 64(10.0%) | 232(36.4%) | 0.003 |
| [V19] I can share my past treatment progress. | 85(13.3%) | 246(38.6%) | 0.348 |
| [V20] Feel free to ask your own questions. | 89(13.9%) | 255(40.0%) | 0.390 |
| [V21] Finding and using the information you need. | 46(7.2%) | 121(19.0%) | 0.927 |
| [V22] Taking action to continue the medication. | 42(6.6%) | 94(14.7%) | 0.287 |
| [V23] Reporting unusual symptoms to health care providers. | 32(5.0%) | 91(14.3%) | 0.728 |
| [V24] None of these (V18-V23) apply to me. | 20(3.1%) | 33(5.2%) | 0.074 |
| I eat three meals every day. | 123(19.3%) | 365(57.2%) | 0.034 |
| Sometimes don't eat breakfast | 40(6.3%) | 73(11.4%) | 0.033 |
| Sometimes don't eat lunch | 18(2.8%) | 31(4.9%) | 0.122 |
| Sometimes don't eat dinner | 6(0.9%) | 10(1.6%) | 0.395 |
| Tablets/Capsules (Dosage forms used) | 161(25.2%) | 448(70.2%) | 0.030 |
| Powdered medicine (Dosage forms used) | 26(4.1%) | 52(8.2%) | 0.200 |
| Tape, Poultice, Plaster (Dosage forms used) | 10(1.6%) | 23(3.6%) | 0.688 |
| Inhaler (Dosage forms used) | 1(0.2%) | 16(2.5%) | 0.052 |
| Ointment (Dosage forms used) | 14(2.2%) | 26(4.1%) | 0.257 |
| Injection (Dosage forms used) | 4(0.6%) | 10(1.6%) | 1.000 |
| Eye drops (Dosage forms used) | 22(3.4%) | 44(6.9%) | 0.243 |
| Nasal drops (Dosage forms used) | 8(1.3%) | 11(1.7%) | 0.141 |
| Others (Dosage forms used) | 3(0.5%) | 2(0.3%) | 0.128 |
| Not taking medication in the morning. | 32(5.0%) | 48(7.5%) | 0.006 |
| Taking medication upon awakening. | 15(2.4%) | 29(4.5%) | 0.293 |
| Taking medicines before breakfast. | 31(4.9%) | 69(10.8%) | 0.362 |
| Taking medicines after breakfast. | 113(17.7%) | 351(55.0%) | 0.007 |
| No midday/afternoon medication. | 121(19.0%) | 316(49.5%) | 0.728 |
| Taking medicines before lunch. | 19(3.0%) | 36(5.6%) | 0.205 |
| Taking medicines after lunch. | 38(6.0%) | 122(19.1%) | 0.248 |
| No evening/nighttime medication. | 48(7.5%) | 162(25.4%) | 0.079 |
| Taking medicines before dinner. | 21(3.3%) | 50(7.8%) | 0.644 |
| Taking medicines after dinner. | 115(18.0%) | 277(43.4%) | 0.140 |
| Gender | 60(9.4%) | 139(21.8%) | 0.272 |

3-2. Univariate analysis: continuous variables （Mann-Whitney U test）

| Variables | Mean (Taking the medication correctly) | Mean (Not taking the medication correctly) | p-value |
| --- | --- | --- | --- |
| Age | 60.46 | 57.47 | 0.009 |
| Duration of using drug. | 3.68 | 3.59 | 0.031 |
| I'm convinced of the necessity of medicine. | 4.36 | 4.14 | <0.001 |
| I think I can't stay healthy without medication. | 3.64 | 3.42 | 0.008 |
| I think I want to take my medicine. | 3.31 | 3.21 | 0.308 |
| I think I want to go off my medicine. | 3.07 | 3.42 | 0.001 |
| Anxious about taking medication. | 2.61 | 2.95 | <0.001 |
| I would like to have my medication reduced. | 3.06 | 3.45 | <0.001 |
| Taking medication is part of my lifestyle, like eating and brushing my teeth. | 4.12 | 3.7 | <0.001 |
| Take the same number and frequency of medicines every day. | 4.43 | 4 | <0.001 |
| Using the drug at approximately the same time each day. | 4.42 | 3.76 | <0.001 |
| Taking meals at approximately the same time each day. | 4.3 | 3.67 | <0.001 |
| Number of drugs prescribed (Total) | 2.89 | 2.72 | 0.182 |
| Number of drugs prescribed (morning) | 2.28 | 1.97 | 0.016 |
| Number of drugs prescribed (midday/afternoon) | 0.56 | 0.52 | 0.705 |
| Number of drugs prescribed (evening/nighttime) | 1.51 | 1.51 | 0.618 |
| Number of drugs prescribed (Before bedtime) | 0.35 | 0.48 | 0.068 |
