# Supplementary material for: Identifying the Relative Importance of Factors Influencing Medication Compliance in General Patients Using Regularized Logistic Regression and LightGBM: Web-Based Survey Analysis
Source: JMIR Form Res. 2024 Dec 23;8:e65882. doi: 10.2196/65882 (PMC11704655; doi:10.2196/65882)
Supplement: Multimedia Appendix 4 [file formative_v8i1e65882_app4.docx]

Table S1 Coefficient values in the process of constructing a logistic regression model. (Variables from regularization model)

|  | 1 | 2 | 3 | 4 | 5 | 6 |
| --- | --- | --- | --- | --- | --- | --- |
| Intercept | -3.0465 | -3.1179 | -2.7383 | -0.8381 | -0.1111 | -0.7142 |
| Type1 diabetes | -1.9732* | -1.945* | -1.9815* | -1.568* | -1.6559* | -1.6951* |
| Hyperlipidemia | -0.4205 | -0.3705 | -0.3497 | -0.2206 | -0.1954 | -0.196 |
| IBD | -29.919 | -29.564 | -29.2089 | -29.6051 | -29.3699 | -29.0654 |
| asthma・COPD | 19.7247 | 22.4601 | 23.9076 | 22.9524 | 11.075 | 21.409 |
| I can share my thoughts and goals. | 0.41 | 0.3762 | 0.3613 | 0.4554* | 0.4425* | 0.4603* |
| Taking action to continue the medication. | -0.3899 | -0.3356 | -0.3507 | -0.4238 | -0.4118 | -0.4201 |
| Tablets/Capsules (Dosage forms used) | 0.2955 | 0.5376 | 0.6587 | 0.7884 |  |  |
| Eye drops (Dosage forms used) | -0.621 | -0.4967 | -0.4987 | -0.2757 | -0.424 | -0.3721 |
| Others (Dosage forms used) | -1.5981 | -1.7287 | -1.8651 | -1.3198 | -1.428 | -1.1985 |
| Not taking medication in the morning. | 0.2066 | 0.2619 | 0.2429 | -0.0816 | -0.0934 | -0.1313 |
| Not using evening/nighttime medication. | 0.3824 | 0.4451 | 0.442 | 0.4236 | 0.4097 | 0.4292 |
| Anxious about taking medications. | -0.1081 | -0.067 | -0.0686 | -0.0847 | -0.0873 | -0.2246* |
| I would like to have my medication reduced. | -0.3898* | -0.4206* | -0.4105* | -0.3106* | -0.2983* |  |
| Taking medication is part of my lifestyle, like eating or brushing my teeth. | 0.103 | 0.1968 | 0.3058* | 0.4985* | 0.4897* | 0.5075* |
| I take the same number and frequency of medicines every day. | 0.1099 | 0.262* |  |  |  |  |
| Using the drug at about the same time each day. | 0.5594* |  |  |  |  |  |
| Taking meals at about the same time each day. | 0.4434* | 0.7083* | 0.7481* |  |  |  |
| number of drugs prescribed (morning) | 0.1713 | 0.1729 | 0.175 | 0.1375 | 0.161 | 0.129 |
| number of drugs prescribed (Before bedtime) | -0.1264 | -0.1045 | -0.1004 | -0.1305 | -0.1461 | -0.162 |

*: p<0.05 (highlighted)

Table S2 Coefficient values in the process of constructing a logistic regression model. (Variables from filter method model)

|  | 1 | 2 | 3 | 4 | 5 | 6 | 7 | 8 | 9 | 10 | 11 | 12 |
| --- | --- | --- | --- | --- | --- | --- | --- | --- | --- | --- | --- | --- |
| Type1 diabetes | -1.6655* | -1.7078* | -1.731* | -1.7713* | -1.4013 | -1.4007 | -1.4059 | -1.4533* | -1.5099* | -1.5051* | -1.3094 | -1.4309* |
| Hypertension | 0.0517 | 0.0798 | 0.0724 | 0.0704 | 0.0772 | 0.0773 | 0.0669 | 0.1351 | 0.1818 | 0.1349 | 0.1443 | 0.1452 |
| Asthma・COPD | 25 | 25.7244 | 31.606 | 17.9104 | 27.6393 | 21.358 | 13.6687 | 28.0498 | 19.5557 | 19.2227 | 8.2486 | 21.8669 |
| I can share my thoughts and goals. | 0.3802 | 0.3513 | 0.3222 | 0.3062 | 0.3309 | 0.3303 | 0.3878 | 0.4077* | 0.3954* | 0.3903* | 0.4305* | 0.4656* |
| Eating three meals every day. | -0.3829 | -0.5168 | -0.4982 | -0.5178 | 0.123 | 0.1229 | 0.0901 | 0.1219 | 0.0995 | 0.1323 | 0.1275 | 0.0967 |
| Sometimes don't eat breakfast | -0.1426 | -0.2668 | -0.2339 | -0.188 | -0.1504 | -0.1509 | -0.203 | -0.2338 | -0.2288 | -0.1551 | -0.1613 | -0.1977 |
| Tablets/Capsules (Dosage forms used) | 0.4889 | 0.6736 | 0.6875 | 0.7985 | 0.8566* | 0.8586* | 0.7487 | 0.7211 |  |  |  |  |
| Inhaler (Dosage forms used) | -0.6281 | -0.6126 | -0.5754 | -0.5118 | -0.2066 | -0.2059 | -0.2682 | -0.2325 | -0.2484 | -0.1937 | -0.1148 | -0.0358 |
| Not taking medication in the morning. | -0.0376 | 0.0119 | 0.0155 | 0.0055 | -0.1618 | -0.1609 | -0.1943 | -0.1758 | -0.1453 | -0.1773 | -0.1559 | -0.1264 |
| Taking medicines after breakfast. | -0.0698 | -0.1129 | -0.1109 | -0.0831 | -0.0364 | -0.0368 | -0.0113 | 0.0084 | 0.0909 | 0.1111 | 0.1361 | 0.1274 |
| No evening/nighttime medication. | 0.4053 | 0.4411 | 0.4565 | 0.4552 | 0.409 | 0.4085 | 0.3279 | 0.3438 | 0.3314 | 0.36 | 0.3126 | 0.3138 |
| Age | -0.0009 | 0.0012 | 0.0008 | 0.0009 | 0.0098 | 0.0099 | 0.0112 |  |  |  |  |  |
| Duration of using drug. | -0.0486 | -0.075 | -0.0707 | -0.0531 | 0.0076 |  |  |  |  |  |  |  |
| I'm convinced of the necessity of medicine. | -0.1422 | -0.1235 |  |  |  |  |  |  |  |  |  |  |
| I think I can't stay healthy without medication. | 0.014 | 0.034 | 0.006 | 0.0075 | 0.0409 | 0.0415 | 0.166 | 0.1505 | 0.1604 | 0.1844 |  |  |
| I think I want to go off my meds. | 0.002 | 0.0045 | 0.0088 | 0.017 | 0.0252 | 0.0252 | -0.0131 | -0.0265 | -0.0322 | -0.1176 | -0.1537 |  |
| Anxious about taking medication. | -0.1304 | -0.0955 | -0.0898 | -0.0918 | -0.0807 | -0.081 | -0.1114 | -0.1195 | -0.1184 | -0.2083* | -0.2066* | -0.271* |
| I would like to have my medication reduced. | -0.3546* | -0.3818* | -0.3778* | -0.3727* | -0.3008* | -0.3005* | -0.286* | -0.2792* | -0.2613* |  |  |  |
| Taking medication is part of my lifestyle, like eating and brushing my teeth. | 0.1441 | 0.2107 | 0.1968 | 0.2985* | 0.4721* | 0.4728* |  |  |  |  |  |  |
| Take the same number and frequency of medicines every d | 0.1342 | 0.267* | 0.2513 |  |  |  |  |  |  |  |  |  |
| Using the drug at about the same time each day. | 0.471* |  |  |  |  |  |  |  |  |  |  |  |
| Taking meals at about the same time each day. | 0.5143* | 0.7484* | 0.7436* | 0.7887* |  |  |  |  |  |  |  |  |
| number of drugs prescribed (morning) | 0.1063 | 0.1133 | 0.1178 | 0.1212 | 0.0611 | 0.0615 | 0.0726 | 0.082 | 0.0867 | 0.059 | 0.0788 | 0.0896 |
| number of drugs prescribed (evening/nighttime) | -0.1564 | -0.135 | -0.133 | -0.132 | -0.1102 | -0.1101 | -0.1177 | -0.1417 | -0.1562 | -0.1726 | -0.1345 | -0.1432 |

*: p<0.05 (highlighted)

Table S3 VIF values in the process of constructing a logistic regression model. (Variables from regularization model)

|  | 1 | 2 | 3 | 4 | 5 | 6 |
| --- | --- | --- | --- | --- | --- | --- |
| Type1 diabetes | 1.0 | 1.0 | 1.0 | 1.0 | 1.0 | 1.0 |
| Hyperlipidemia | 1.3 | 1.3 | 1.3 | 1.3 | 1.3 | 1.3 |
| IBD | 1.3 | 1.3 | 1.3 | 1.3 | 1.3 | 1.3 |
| asthma・COPD | 1.0 | 1.0 | 1.0 | 1.0 | 1.0 | 1.0 |
| I can share my thoughts and goals. | 2.1 | 2.1 | 2.1 | 2.0 | 2.0 | 2.0 |
| Taking action to continue the medication. | 1.4 | 1.4 | 1.4 | 1.3 | 1.3 | 1.3 |
| Tablets/Capsules (Dosage forms used) | 20.3 | 19.9 | 18.4 | **16.7** |  |  |
| Eye drops (Dosage forms used) | 1.3 | 1.3 | 1.3 | 1.2 | 1.2 | 1.2 |
| Others (Dosage forms used) | 1.3 | 1.3 | 1.3 | 1.3 | 1.3 | 1.3 |
| Not taking medication in the morning. | 1.7 | 1.7 | 1.7 | 1.7 | 1.7 | 1.7 |
| Not using evening/nighttime medication. | 1.6 | 1.6 | 1.6 | 1.6 | 1.6 | 1.6 |
| Anxious about taking medications. | 10.3 | 10.2 | 10.2 | 10.2 | 9.9 | 5.7 |
| I would like to have my medication reduced. | 13.8 | 13.6 | 13.6 | 13.4 | **12.6** |  |
| Taking medication is part of my lifestyle, like eating or brushing my teeth. | 30.6 | 29.1 | 21.7 | 14.9 | 10.2 | 9.3 |
| I take the same number and frequency of medicines every day. | 41.0 | **35.6** |  |  |  |  |
| Using the drug at about the same time each day. | **68.8** |  |  |  |  |  |
| Taking meals at about the same time each day. | 41.8 | 25.1 | **22.6** |  |  |  |
| number of drugs prescribed (morning) | 5.3 | 5.3 | 5.3 | 5.3 | 5.1 | 4.9 |
| number of drugs prescribed (Before bedtime) | 1.4 | 1.4 | 1.4 | 1.3 | 1.3 | 1.3 |

The value with the highest VIF in each row is considered to be experiencing multicollinearity and is removed from the model. Variables that were removed are highlighted.

Table S4 VIF values in the process of constructing a logistic regression model. (Variables from filter method model)

|  | 1 | 2 | 3 | 4 | 5 | 6 | 7 | 8 | 9 | 10 | 11 | 12 |
| --- | --- | --- | --- | --- | --- | --- | --- | --- | --- | --- | --- | --- |
| Type1 diabetes | 1.1 | 1.1 | 1.1 | 1.1 | 1.1 | 1.1 | 1.1 | 1.1 | 1.0 | 1.0 | 1.0 | 1.0 |
| Hypertension | 2.2 | 2.2 | 2.2 | 2.2 | 2.2 | 2.2 | 2.2 | 2.1 | 2.0 | 2.0 | 2.0 | 2.0 |
| Asthma・COPD | 2.3 | 2.3 | 2.3 | 2.3 | 2.3 | 2.3 | 2.3 | 2.3 | 2.3 | 2.3 | 2.3 | 2.3 |
| I can share my thoughts and goals. | 2.1 | 2.1 | 2.0 | 2.0 | 2.0 | 2.0 | 2.0 | 2.0 | 2.0 | 2.0 | 1.9 | 1.9 |
| I eat three meals every day. | 14.2 | 14.1 | 14.0 | 14.0 | 13.1 | 13.0 | 12.7 | 11.4 | 10.8 | 10.7 | 9.2 | 8.3 |
| Sometimes don't eat breakfast | 3.8 | 3.7 | 3.7 | 3.7 | 3.7 | 3.7 | 3.6 | 3.4 | 3.2 | 3.2 | 2.8 | 2.5 |
| Tablets/Capsules (Dosage forms used) | 23.0 | 22.6 | 22.4 | 21.7 | 21.4 | 20.7 | 20.4 | **19.3** |  |  |  |  |
| Inhaler (Dosage forms used) | 2.3 | 2.3 | 2.3 | 2.3 | 2.3 | 2.3 | 2.3 | 2.3 | 2.3 | 2.3 | 2.3 | 2.3 |
| Not taking medication in the morning. | 2.4 | 2.3 | 2.3 | 2.3 | 2.3 | 2.3 | 2.3 | 2.3 | 2.2 | 2.2 | 2.0 | 2.0 |
| Taking medicines after breakfast. | 6.5 | 6.5 | 6.5 | 6.5 | 6.5 | 6.5 | 6.4 | 6.4 | 5.9 | 5.9 | 5.6 | 5.5 |
| No evening/nighttime medication. | 1.7 | 1.7 | 1.7 | 1.7 | 1.7 | 1.7 | 1.7 | 1.7 | 1.6 | 1.6 | 1.6 | 1.6 |
| Age | 30.1 | 30.0 | 28.5 | 28.4 | 27.0 | 23.8 | **22.0** |  |  |  |  |  |
| Duration of using drug. | 30.6 | 30.6 | 30.5 | 30.3 | **30.0** |  |  |  |  |  |  |  |
| I'm convinced of the necessity of medicine. | 54.4 | **54.2** |  |  |  |  |  |  |  |  |  |  |
| I think I can't stay healthy without medication. | 22.1 | 22.1 | 18.8 | 18.8 | 18.7 | 18.2 | 14.2 | 13.8 | 12.1 | **12.1** |  |  |
| I think I want to go off my meds. | 13.9 | 13.9 | 13.8 | 13.7 | 13.7 | 13.6 | 13.6 | 13.5 | 13.4 | 10.9 | **10.9** |  |
| Anxious about taking medication. | 11.3 | 11.2 | 11.2 | 11.2 | 11.2 | 11.2 | 11.2 | 11.2 | 11.2 | 9.3 | 9.1 | 6.1 |
| I would like to have my medication reduced. | 16.8 | 16.6 | 16.6 | 16.6 | 16.6 | 16.5 | 16.5 | 16.2 | **15.8** |  |  |  |
| Taking medication is part of my lifestyle, like eating and brushing my teeth. | 37.7 | 36.4 | 34.6 | 28.8 | 25.5 | **24.4** |  |  |  |  |  |  |
| Take the same number and frequency of medicines every day. | 44.6 | 39.1 | **37.6** |  |  |  |  |  |  |  |  |  |
| Using the drug at about the same time each day. | **69.7** |  |  |  |  |  |  |  |  |  |  |  |
| Taking meals at about the same time each day. | 50.0 | 32.9 | 32.7 | **30.4** |  |  |  |  |  |  |  |  |
| number of drugs prescribed (morning) | 5.8 | 5.8 | 5.7 | 5.7 | 5.7 | 5.6 | 5.6 | 5.5 | 5.5 | 5.3 | 4.9 | 4.9 |
| number of drugs prescribed (evening/nighttime) | 1.5 | 1.5 | 1.5 | 1.5 | 1.5 | 1.5 | 1.5 | 1.4 | 1.4 | 1.4 | 1.4 | 1.4 |
