# Supplementary material for: Identifying the Relative Importance of Factors Influencing Medication Compliance in General Patients Using Regularized Logistic Regression and LightGBM: Web-Based Survey Analysis
Source: JMIR Form Res. 2024 Dec 23;8:e65882. doi: 10.2196/65882 (PMC11704655; doi:10.2196/65882)
Supplement: Multimedia Appendix 5 [file formative_v8i1e65882_app5.docx]

Table S1 regularization model

| Features | Coefficient [95%CI] | p-value |
| --- | --- | --- |
| Type1 diabetes | -1.9538 [-4.5782, 0.813] | 0.088 |
| Hyperlipidemia | -0.3866 [-0.9228, 0.0852] | 0.132 |
| I can share my thoughts and goals. | 0.3671 [-0.1645, 0.8387] | 0.196 |
| Taking action to continue the medication. | -0.4316 [-1.0334, 0.1791] | 0.172 |
| Inhaler (Dosage forms used) | 2.3467 [-6.8305, 4.3721] | 1.000 |
| Eye drops (Dosage forms used) | -0.7362 [-1.4088, -0.0851] | 0.024* |
| Not taking medication in the morning. | 0.1308 [-0.6102, 0.944] | 0.744 |
| No evening/nighttime medication. | 0.3473 [-0.1718, 0.7763] | 0.200 |
| I think I want to go off my medicine. | -0.0146 [-0.2515, 0.2062] | 0.892 |
| Anxious about taking medication. | -0.1109 [-0.3955, 0.2032] | 0.384 |
| I would like to have my medication reduced. | -0.3207 [-0.5967, 0.0181] | 0.080 |
| Taking medication is part of my lifestyle, like eating and brushing my teeth. | 0.1359 [-0.1917, 0.4791] | 0.480 |
| Take the same number and frequency of medicines every day. | 0.0962 [-0.1585, 0.4653] | 0.484 |
| Using the drug at approximately the same time each day. | 0.5629 [0.1152, 0.9041] | 0.008** |
| Taking meals at approximately the same time each day. | 0.3889 [0.0432, 0.7361] | 0.036* |
| Number of drugs prescribed (morning) | 0.1443 [-0.0576, 0.3046] | 0.184 |
| Number of drugs prescribed (Before bedtime) | -0.143 [-0.4051, 0.1249] | 0.264 |

*: p<0.05, **: p<0.01

Table S2 filter method model

| Features | Coefficient [95%CI] | p-value |
| --- | --- | --- |
| Type1 diabetes | -1.6521 [-3.156, -0.149] | 0.031 * |
| Hypertension | 0.0236 [-0.428, 0.475] | 0.918 |
| I can share my thoughts and goals. | 0.3568 [-0.061, 0.775] | 0.094 |
| Eating three meals every day. | -0.3519 [-1.218, 0.515] | 0.426 |
| Sometimes don't eat breakfast | -0.1046 [-1.037, 0.828] | 0.826 |
| Tablets/Capsules (Dosage forms used) | 0.5036 [-0.419, 1.426] | 0.285 |
| Inhaler (Dosage forms used) | 2.4334 [0.054, 4.813] | 0.045 |
| Not taking medication in the morning. | -0.0579 [-0.87, 0.754] | 0.889 |
| Taking medicines after breakfast. | -0.051 [-0.614, 0.512] | 0.859 |
| Age | -0.0021 [-0.02, 0.016] | 0.817 |
| No evening/nighttime medication. | 0.4002 [-0.063, 0.863] | 0.09 |
| Duration of using drug. | -0.0696 [-0.358, 0.219] | 0.636 |
| I'm convinced of the necessity of medicine. | -0.1294 [-0.482, 0.224] | 0.472 |
| I think I can't stay healthy without medication. | 0.0172 [-0.232, 0.267] | 0.892 |
| I think I want to go off my medicine. | -0.0008 [-0.237, 0.235] | 0.995 |
| Anxious about taking medication. | -0.1252 [-0.356, 0.106] | 0.288 |
| I would like to have my medication reduced. | -0.3371 [-0.587, -0.087] | 0.008** |
| Taking medication is part of my lifestyle, like eating and brushing my teeth. | 0.173 [-0.111, 0.457] | 0.232 |
| Take the same number and frequency of medicines every day. | 0.1169 [-0.168, 0.402] | 0.421 |
| Using the drug at approximately the same time each day. | 0.4599 [0.109, 0.811] | 0.01* |
| Taking meals at approximately the same time each day. | 0.4914 [0.174, 0.808] | 0.002** |
| Number of drugs prescribed (morning) | 0.0964 [-0.087, 0.28] | 0.302 |
| Number of drugs prescribed (Before bedtime) | -0.1624 [-0.406, 0.082] | 0.192 |

*: p<0.05, **: p<0.01
